# Immune effector cell-associated neurotoxicity syndrome: integrative mechanisms, predictive biomarkers, and translational pathways for prevention in CAR T-cell therapy

**DOI:** 10.3389/fneur.2026.1739021

**Published:** 2026-03-04

**Authors:** Faisal Aziz, Abhijit Chakraborty, Dwaipayan Saha, Preyangsee Dutta

**Affiliations:** 1 The Hormel Institute, University of Minnesota, Austin, MN, United States; 2 CellGene Nexus, Rosharon, TX, United States; 3 Department of Investigational Cancer Therapeutics, The University of Texas MD Anderson Cancer Center, Houston, TX, United States; 4 Metabolic, Nutrition and Exercise Research (MiNER) Laboratory, The University of Texas at El Paso, El Paso, TX, United States

**Keywords:** biomarker framework, CAR T-cell therapy, endothelial dysfunction, immune effector cell-associated neurotoxicity (ICANS), neuroinflammation, translational neurotherapeutics

## Abstract

Immune effector cell-associated neurotoxicity syndrome (ICANS) is a common and sometimes severe complication of chimeric antigen receptor (CAR) T-cell therapy. Although our understanding has advanced considerably, ICANS remains biologically complex and clinically variable. In this review, we synthesize current evidence on how systemic immune activation, endothelial injury, disruption of the blood–brain barrier, and neuroinflammation converge to produce neurological symptoms in affected patients. We summarize emerging predictive biomarkers across plasma, cerebrospinal fluid, electroencephalography (EEG), and neuroimaging, and organize them within a temporal framework to highlight when different signals arise and how they may support earlier recognition. We also differentiate ICANS from tumor inflammation–associated neurotoxicity (TIAN), a syndrome more frequently observed in patients with central nervous system tumors, underscoring key differences in pathogenesis, presentation, and management. Finally, we discuss conceptual approaches to multimodal risk prediction and the practical challenges that currently limit clinical implementation, including assay turnaround time, generalizability across CAR constructs and disease settings, interpretability, and ethical considerations when acting on predicted risk. We propose a pragmatic roadmap that prioritizes prospective biomarker-guided studies, standardized assay platforms, and transparent modeling strategies to help move the field from observation toward safer prevention. Taken together, this integrative perspective aims to clarify the biology of ICANS, contextualize emerging biomarkers, and support more informed and safer use of CAR T-cell therapy.

## 1 Introduction

CAR T-cell therapy has provided durable remissions in many patients with relapsed or refractory hematologic malignancies; however, this remarkable clinical benefit frequently occurs alongside immune-mediated toxicities, principally cytokine release syndrome (CRS) and ICANS. Incidence estimates vary according to product characteristics, underlying diseases, patient selection criteria, and grading systems. Pooled analyses revealed that approximately 25% of patients developed ICANS of any grade, while approximately 10% experienced severe or high-grade neurotoxicity. Notably, certain cohort studies have reported higher incidence rates depending on the CAR construct design and patient characteristics. ICANS typically emerges within 2–12 days post-infusion, with clinical manifestations ranging from mild cognitive impairment and aphasia to seizures, cerebral edema, and, in severe cases, fatal encephalopathy. A subset of patients may also experience long-term or persistent neurological sequelae (1, 2).

This review focuses on Immune Effector Cell-Associated Neurotoxicity Syndrome (ICANS), the neurotoxicity syndrome most commonly observed following CAR T-cell therapy for hematologic malignancies. It is critical to distinguish ICANS from other neuroinflammatory conditions, such as Tumor Inflammation-Associated Neurotoxicity (TIAN), which occurs in patients with central nervous system (CNS) tumors receiving local immunotherapies (3–7). While both involve neuroinflammation, TIAN reflects tumor-localized inflammatory edema and increased intracranial pressure, whereas ICANS is characterized by a more diffuse encephalopathy driven by systemic cytokine signaling and blood–brain barrier disruption (6). The predictive biomarkers, mechanistic cascades, and prophylactic strategies discussed herein are primarily framed within the context of ICANS, acknowledging that the neurotoxicity landscape will evolve as CAR T-cell therapy expands to new anatomical and disease settings (8).

Several studies have described different aspects of the ICANS pathway, including cytokines, endothelial dysfunction, BBB disruption, and clinical management. However, only a few have tried to connect these findings across scales or propose operational predictive pipelines that combine molecular data, imaging, electrophysiology and mechanistic modeling (9–12). Most biomarker studies are based on small, single-center cohorts with inconsistent sampling times, assay methods, and clinical criteria. This inconsistency makes it difficult to compare studies and validate the predictive models. There is an urgent need for a comprehensive review that connects molecular mechanisms to patient outcomes, organizes potential biomarkers within specific time windows, and outlines practical modeling and trial strategies to translate predictive biomarkers into prophylactic strategies (4, 13–17).

In this review, we thus aim to present a multi-scale mechanistic framework for ICANS, curate and classify candidate biomarkers based on sample source and temporal window, summarize therapeutic and prophylactic approaches with mechanistic rationale and supporting evidence, and propose a modular predictive modeling pipeline that outlines a translational roadmap for biomarker-guided ICANS mitigation (18–23).

## 2 Pathophysiology: a multi-scale view

The complexities of ICANS necessitate a comprehensive understanding of multiple biological processes involved in its pathogenesis. This framework integrates cytokine-mediated molecular signaling, glial-immune cellular interplay, and higher-order neurovascular and glymphatic dynamics, which collectively orchestrate blood–brain barrier integrity and CNS homeostasis. These micro to macro-scale interactions directly correlate with clinical manifestations observed in patients, such as seizures and encephalopathy, thus forming a layered approach to understanding ICANS (24, 25).

### 2.1 Molecular and cellular mechanisms

ICANS arises from a connected series of immune, vascular, and neural events in which cytokine release, endothelial injury, and glial activation interact across molecular to whole-body levels, causing brain toxicity. Activated CAR T cells proliferate rapidly and secrete large quantities of pro-inflammatory cytokines such as interleukin-6 (IL-6), interleukin-1 (IL-1), interferon-*γ* (IFN-γ), and tumor necrosis factor-*α* (TNF-α). This systemic cytokine surge drives a self-amplifying inflammatory loop that precedes both cytokine release syndrome (CRS) and ICANS (26–33).

The inflammatory milieu drives widespread endothelial activation, characterized by the upregulation of adhesion molecules and the release of Weibel–Palade body constituents, such as von Willebrand factor (vWF) and Angiopoietin-2 (Ang-2). Elevated Ang-2 levels signal endothelial destabilization and correlate with increased microvascular permeability and BBB disruption, thereby permitting immune mediators to enter the CNS (34). Sustained endothelial injury has been associated with the severity of CAR T-cell–related toxicities (27, 35–39).

Cytokine-driven inflammation also activates complement and coagulation cascades, generating reactive oxygen species (ROS) that exacerbate endothelial and parenchymal injuries. These processes link systemic cytokine storms to localized neuroinflammatory injuries. Within the CNS, resident microglia and astrocytes amplify inflammatory signaling and modulate glutamate homeostasis while maintaining the BBB structure. Elevated levels of glial fibrillary acidic protein (GFAP) and neurofilament light chain (NfL) during ICANS episodes reflect glial activation and neuronal injury (40–48).

Inflammation further disrupts tight junction proteins including claudins and occludins, thus compromising BBB integrity (4, 49–55) and facilitating cytokine entry into the cerebrospinal fluid (CSF) and parenchyma (56–61). Patients with severe neurotoxicity frequently exhibit elevated CSF cytokine levels that correlate with clinical severity (27, 62).

**Figure 1:**
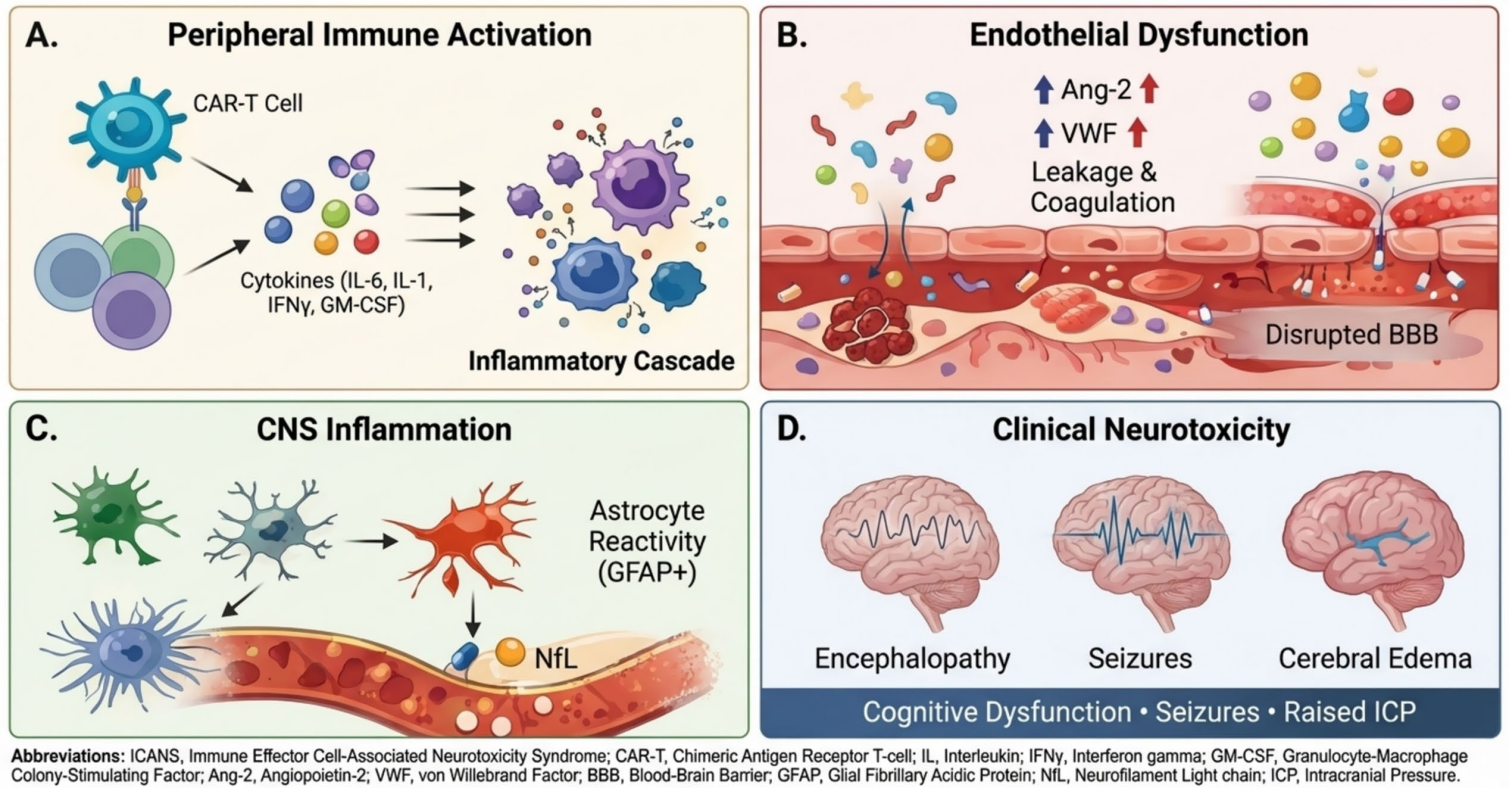
Mechanistic overview of ICANS following CAR T-cell therapy (schematic). This schematic summarizes major biological processes implicated in immune effector cell-associated neurotoxicity syndrome (ICANS). **(A)** CAR T-cell activation triggers systemic immune activation and cytokine release. **(B)** Elevated inflammatory mediators and endothelial activation (e.g., Ang-2 and von Willebrand factor) contribute to microvascular injury and blood–brain barrier disruption. **(C)** Entry of inflammatory mediators into the central nervous system promotes astrocyte reactivity and neuronal injury, reflected by increases in glial and axonal biomarkers such as GFAP and neurofilament light chain. **(D)** These processes culminate in clinical neurotoxicity, including encephalopathy, seizures, and, in severe cases, cerebral edema.

Collectively, these processes form a multi-scale cascade linking CAR T-cell activation, cytokine release, endothelial dysfunction, and CNS inflammation to the clinical manifestations of ICANS. To provide a conceptual framework for interpreting downstream biomarkers and translational data, we first summarize the major biological processes implicated in ICANS. Current evidence suggests that systemic cytokine release, endothelial activation, disruption of the BBB, and subsequent CNS inflammation interact to produce the spectrum of neurological manifestations observed after CAR T-cell therapy (Figure 1). These processes likely occur in overlapping and patient-specific patterns rather than as a single linear cascade.

### 2.2 Systems and spatial dynamics

Systemic cytokine gradients and endothelial dysfunction promote the migration of inflammatory mediators into the perivascular spaces within the CNS. These processes, coupled with impaired glymphatic clearance, create a permissive environment for sustained inflammation and neuronal injury (55, 63–66).

Neuroimaging and histopathological studies highlight regional vulnerabilities, with pronounced susceptibility of the deep gray nuclei, hippocampus, and subcortical white matter. Perfusion imaging often reveals hypoperfusion and metabolic stress in affected regions, indicating cerebral autoregulatory failure (55, 67–72).

Disruption of the glymphatic system impairs the clearance of neurotoxic solutes, aggravating cytokine retention and increasing neuronal stress. Although clinical validation remains limited, preclinical models suggest that impaired glymphatic flow prolongs CNS inflammation during ICANS (4, 73–77). Inflammation-induced endothelial dysfunction compromises cerebral autoregulation, resulting in hypoperfusion and microvascular shunting. These alterations may synergize with excitotoxic processes to disrupt neuronal metabolism and structural integrity, providing a mechanistic explanation for MRI abnormalities observed in severe ICANS (78–83).

### 2.3 Translational and human observations

Although human autopsy data remain scarce, reported cases consistently demonstrate microvascular injury, petechial hemorrhages, and perivascular inflammation, supporting the BBB-centered microvascular pathology underlying ICANS (84, 85). Magnetic resonance imaging (MRI) findings in ICANS range from normal scans in mild cases to marked T2/FLAIR hyperintensities, diffusion restrictions, and perfusion deficits in severe cases. These imaging features provide mechanistic and diagnostic insight, complementing ongoing efforts by multicenter consortia to standardize neuroimaging in CAR T-cell neurotoxicity (55, 86, 87).

In both serum and CSF, elevated pro-inflammatory cytokine levels (IL-6, IL-1β, GM-CSF) correlated with ICANS and CRS severity. Markers of endothelial activation (Ang-2, vWF) and neuronal injury (NfL, GFAP) show dynamic changes that parallel clinical progression, though further longitudinal validation is needed (34, 83, 88–90). Electroencephalography (EEG) frequently reveals diffuse slowing, delta-theta dominance, and occasional epileptiform discharges in patients with ICANS. Quantitative EEG metrics have emerged as potential early indicators of neurotoxicity risk, however, larger, prospective cohorts are required to confirm their predictive utility (91–97).

## 3 Biomarkers and predictors of ICANS risk

Evaluating biomarkers for immune effector cell-associated neurotoxicity syndrome (ICANS) is critical for early risk stratification, patient monitoring, and timely therapeutic intervention after CAR T-cell therapy. These biomarkers can be categorized temporally into three stages: pre-infusion (baseline risk stratifiers), early on-treatment (dynamic predictors), and post-treatment (indicators of injury). This structured framework integrates data from plasma, cerebrospinal fluid (CSF), imaging modalities, and electroencephalography (EEG), emphasizing the need for multimodal assessment supported by variable levels of evidence across studies (98).

**Figure 2:**
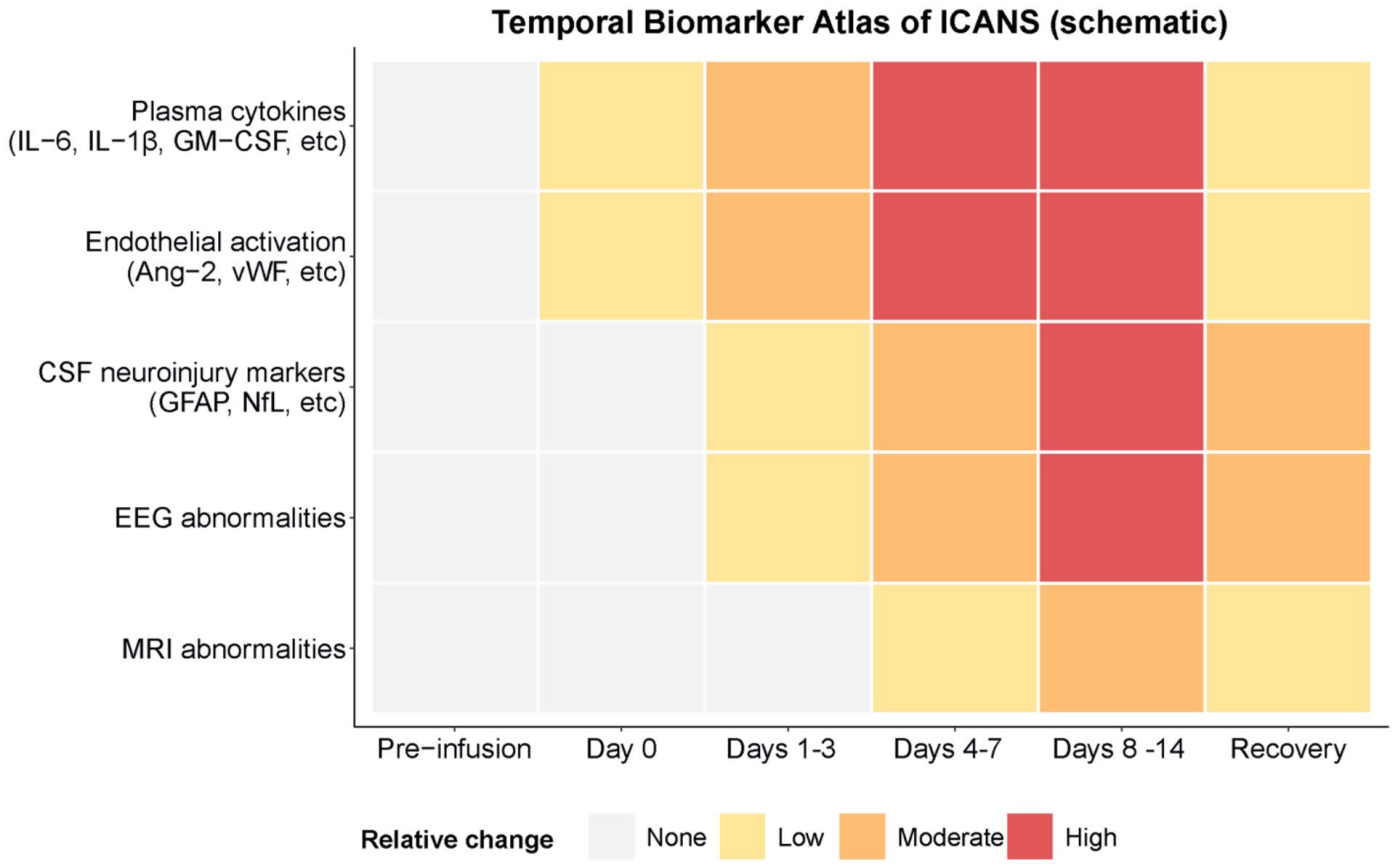
Temporal biomarker atlas of ICANS. This schematic heatmap summarizes the temporal patterns of candidate biomarkers reported in immune effector cell-associated neurotoxicity syndrome (ICANS) following CAR T-cell infusion. Biomarkers are organized by source (plasma, cerebrospinal fluid, EEG, and MRI) and displayed across clinically relevant time windows. Color intensity reflects relative change from baseline and is intended to illustrate qualitative trends rather than quantitative values. Early elevations in plasma cytokines and endothelial markers define a predictive period preceding neurological symptoms, followed by later increases in CSF markers and electrophysiologic abnormalities during the diagnostic phase. Imaging and neurophysiologic abnormalities may persist into the recovery period. This conceptual framework highlights mechanistic windows that may support biomarker-guided monitoring and early intervention strategies. To illustrate how multimodal biomarkers might ultimately be incorporated into clinical decision-making, we developed a conceptual integration framework. In this hypothesis-generating model, clinical risk factors, plasma biomarkers, EEG findings, and imaging are combined within a weighted integration layer that could incorporate both mechanistic knowledge and data-driven approaches. Rather than proposing a deployable tool, this framework highlights how multimodal information might eventually support risk stratification, guide monitoring intensity, and inform prophylactic strategies in prospective studies.

To better synthesize candidate biomarkers across plasma, cerebrospinal fluid, electrophysiologic, and imaging modalities, we developed a schematic “Temporal Biomarker Atlas” that summarizes their relative patterns following CAR T-cell infusion (Figure 2). This representation is conceptual and is intended to highlight qualitative temporal relationships rather than precise quantitative changes. Early increases in plasma cytokines and endothelial markers (IL-6, IL-1β, GM-CSF, Ang-2, and vWF) define a predictive phase that precedes the onset of neurotoxicity. During the diagnostic phase (Days 7–14), cerebrospinal fluid biomarkers such as GFAP and NfL increase in parallel with EEG slowing and MRI abnormalities, reflecting astroglial and neuronal injury. The delayed recovery phase is marked by the gradual normalization of electrophysiological and imaging findings. This temporal framework highlights mechanistic windows that can inform biomarker-guided risk stratification and early intervention strategies for ICANS.

### 3.1 Pre-infusion risk factors

Baseline clinical and biological features assessed before CAR-T cell infusion can help identify patients at a higher risk of treatment-related neurotoxicity. Several clinical parameters have been associated with an increased ICANS risk, including a high tumor burden, specific disease types such as acute lymphoblastic leukemia (ALL) compared with non-Hodgkin lymphoma (NHL), and pre-existing central nervous system (CNS) involvement. The degree of lymphodepletion and the total CAR T-cell dose administered have also been correlated with the likelihood of developing ICANS (8, 33, 99–103).

Baseline biomarker profiling provides additional insights into patients’ vulnerabilities. Elevated pre-infusion levels of inflammatory markers such as C-reactive protein (CRP) and ferritin, together with endothelial injury indicators like Angiopoietin-2 (Ang-2) and von Willebrand factor (vWF), have been linked to post-infusion neurotoxicity. Biomarkers of neuronal and glial integrity, including neurofilament light chain (NfL) and glial fibrillary acidic protein (GFAP), may also reveal pre-existing neuroanatomical susceptibility in patients with a history of neurological compromise. Electroencephalographic abnormalities such as elevated global abnormality scores and diffuse slowing have been associated with a pre-symptomatic state of cerebral vulnerability that increases the likelihood of ICANS (104–108).

### 3.2 Early on-treatment biomarkers

Dynamic changes in cytokines, endothelial markers, and neurophysiological parameters within the first 2 weeks after infusion are among the most informative predictors of ICANS. Serial monitoring of cytokine kinetics, including interleukin-6 (IL-6), interleukin-1 (IL-1), and granulocyte-macrophage colony-stimulating factor (GM-CSF), has shown that both the peak concentration and rate of cytokine elevation are associated with neurotoxicity severity. Frequent sampling during the first 14 days post-infusion helps characterize these trajectories and their relationship to clinical onset (33, 109–113).

Endothelial injury markers add an important vascular dimension to the risk prediction. Elevated Ang-2, a higher Ang-2 to Ang-1 ratio and increased soluble thrombomodulin are all indicative of endothelial stress and microvascular destabilization that can lead to blood–brain barrier (BBB) disruption (83, 114–116). Neuroimaging has further expanded the early predictive capabilities. Magnetic resonance imaging (MRI) studies have shown that diffusion-weighted imaging (DWI) and apparent diffusion coefficient (ADC) abnormalities can appear several days before clinical symptoms. Imaging performed between days three and seven post-infusion may reveal vasogenic or cytotoxic edema, helping identify patients at risk for developing ICANS (55, 117–121).

Quantitative EEG is a complementary functional tool. Reductions in background frequency, increased delta-theta power, and disrupted network connectivity patterns have been correlated with ICANS severity. Continuous EEG monitoring and derived composite scores are being evaluated as real-time predictive markers in high-risk patients (97, 122–125).

Although invasive, CSF analysis provides valuable insights into intrathecal inflammation and BBB compromise. Elevated cytokine, GFAP, and NfL concentrations in CSF parallel systemic inflammatory changes and correlate with clinical severity, reinforcing the multi-compartmental nature of ICANS pathophysiology (126–132).

### 3.3 Multimodal risk models

Recent studies have attempted to integrate clinical, biochemical, imaging, and electrophysiological markers into a unified predictive framework. Composite indices that combine cytokine kinetics, endothelial markers, and neurophysiological parameters show improved predictive value compared to individual markers, although most models remain limited to single-center or small cohort studies. Larger multicenter efforts and standardized validation protocols are necessary to establish reproducibility and clinical reliability (133–138). Emerging models emphasize the potential of multimodal integration. Linking temporal cytokine profiles with imaging abnormalities and EEG signatures may enhance early risk prediction and allow more proactive management of neurotoxicity. Future research should focus on harmonized sampling schedules, diverse patient populations, and the application of machine learning algorithms to develop real-time adaptive prediction systems. Such advances will be crucial for translating biomarker discovery into practical tools that improve patient safety and treatment outcomes in CAR-T cell therapy.

## 4 Computational and predictive modeling approaches

Predicting and understanding immune effector cell-associated neurotoxicity syndrome (ICANS) is a multiscale challenge that requires the integration of biological mechanisms and computational methods. The pathophysiology of ICANS includes molecular signaling, cellular kinetics, tissue-level barrier dysfunction, and systemic manifestations. To capture these interconnected layers, modeling frameworks must represent both underlying biology and emergent clinical outcomes. Hybrid computational approaches that combine mechanistic ordinary differential equation (ODE) models with contemporary machine learning (ML) techniques have emerged as promising tools for improving predictive power while maintaining biological interpretability (22, 139–141).

### 4.1 Current state of computational modeling in CAR T-cell therapy

Most existing computational studies on CAR T-cell therapy focus on cellular expansion kinetics, tumor burden dynamics, biodistribution, and pharmacokinetic or pharmacodynamic (PK/PD) relationships that describe CAR T-cell trafficking and persistence. Systematic reviews and semi-mechanistic models have established a strong foundation for understanding these behaviors *in vivo* and *in vitro*. These models typically employ ODE systems that capture CAR T-cell proliferation, tumor-immune interactions, compartmental trafficking, and cytokine-mediated feedback (4, 142–146).

CAR T-cell proliferation is often modeled as antigen-driven expansion with logistic constraints, whereas tumor-immune interactions are described using predator–prey dynamics. Multicompartmental models are used to simulate cell distribution across the blood, lymphoid organs, bone marrow, and tumor sites. Cytokine feedback loops, including IL-2 and IL-6 signaling, are frequently incorporated to reflect their modulatory effects on CAR-T cell activation and persistence.

However, models specifically designed to predict neurotoxicity remain scarce. The few existing approaches are limited by small sample sizes, a lack of mechanistic grounding, minimal incorporation of neurological biomarkers, and insufficient temporal resolution. These gaps underscore the need for models validated with longitudinal, multimodal datasets that capture the full trajectory of neurotoxicity from subclinical changes to overt ICANS (147, 148).

### 4.2 Extending modeling frameworks to neurotoxicity

A comprehensive computational framework for ICANS prediction should integrate mechanistic modeling, probabilistic assessment, and data-driven learning. These components operate at complementary biological and computational scales.

#### 4.2.1 Mechanistic ODE modules for cytokine kinetics and endothelial stress

These modules represent the temporal evolution of cytokines implicated in ICANS, including IL-6, IL-1, IFN-*γ*, TNF-*α*, IL-15, and GM-CSF levels. The ODE systems capture cytokine production from CAR T cells, myeloid cells, and endothelial sources, as well as clearance via receptor-mediated uptake and degradation. State variables may include endothelial activation markers, such as ICAM-1, VCAM-1, and E-selectin. Positive feedback through IL-6 trans-signaling and negative regulation through IL-10 or regulatory T cells can be encoded to represent a dynamic balance. Population-level parameters can be estimated using nonlinear mixed-effects modeling, which accounts for inter-patient variability and enables individualized predictions.

#### 4.2.2 Probabilistic blood–brain barrier rupture module

This module quantifies the likelihood of BBB compromise based on the inflammatory and endothelial injury markers. It can use threshold- or regression-based formulations to predict BBB permeability when weighted biomarker combinations surpass a critical limit. The framework may incorporate clinical covariates, such as hypertension or diabetes, which influence BBB vulnerability. The model outputs a time-dependent probability of BBB disruption, which can serve as an input for downstream CNS exposure models.

#### 4.2.3 Diffusion and compartmental CNS models

These models simulate cytokine transport from the systemic circulation to the CNS, linking the plasma and CSF compartments through physiological permeability relationships. They distinguish blood, ventricular, lumbar CSF, and interstitial fluid spaces, and allow the permeability coefficients to vary dynamically with BBB status. Integration of perivascular (glymphatic) flow and regional vulnerability mapping can further improve realism, especially in anatomically sensitive brain regions.

#### 4.2.4 Supervised machine learning classification module

This layer integrates the outputs of mechanistic models with clinical and biomarker data to generate predictive outcomes. Algorithms such as random forests, gradient boosting, and deep neural networks can be trained using cytokine trajectories, endothelial stress indices, imaging features, and EEG-derived parameters. Predictions may include binary ICANS occurrence, graded severity, time-to-onset, and distinct phenotypes, such as encephalopathy or cerebral edema. Mechanistic outputs serve as biologically meaningful features that improve interpretability and reduce overfitting compared with black-box ML models (147, 148).

#### 4.2.5 Intervention simulation

Hybrid models also support in silico simulations of therapeutic interventions. They can estimate the effects of anti-cytokine therapies such as tocilizumab, siltuximab, or anakinra, evaluate corticosteroid timing, and explore prophylactic versus reactive treatment strategies (149, 150). These simulations can guide clinical trial design and personalized treatment optimization (147, 148).

### 4.3 Time-series modeling techniques for longitudinal biomarker streams

The dynamic nature of CAR T-cell responses necessitates time-series modeling to accurately characterize the evolving biomarker profiles. Recurrent neural networks (RNNs), including long short-term memory (LSTM) and gated recurrent unit (GRU) architectures, are particularly effective for modeling sequential dependencies in cytokine and electroencephalogram (EEG) data. Bidirectional RNNs and attention mechanisms can improve the predictive accuracy by emphasizing temporally relevant features.

Temporal convolutional networks (TCNs) offer an alternative approach that efficiently captures long-range dependencies and multiscale temporal dynamics. These models are computationally efficient and suitable for real-time monitoring.

State-space and Kalman filter models provide probabilistic tracking of latent disease states, accommodating noisy and irregularly sampled data. Bayesian hierarchical models extend these concepts by allowing the estimation of individual-level trajectories within a population framework. Informative priors derived from preclinical data or related immunotherapies can reduce overfitting and provide uncertainty estimates useful for clinical decision-making (97, 151–156).

### 4.4 Spatial and agent-based modeling paradigms

Spatially explicit models provide insights into mechanistic processes that cannot be captured using purely temporal approaches. Agent-based models (ABMs) simulate interactions between individual cells, such as CAR T cells, tumor cells, endothelial cells, and microglia. Each agent follows biologically defined rules that govern migration, proliferation, cytotoxicity, and cytokine secretion. These simulations can replicate regional vulnerabilities within the CNS and capture the emergent behaviors that lead to neuroinflammatory amplification.

Partial differential equation (PDE) models complement ABMs by describing continuous cytokine diffusion and spatial gradients in the tissue. Hybrid ABM-PDE systems combine discrete cellular behaviors with continuous molecular fields, maintaining mechanistic detail while improving computational efficiency.

Although spatial models are valuable for hypothesis generation and preclinical research, their clinical translation remains challenging; their greatest utility lies in hypothesis testing and in identifying key biological drivers that can inform simpler predictive models (157–161).

### 4.5 Case studies, prototype models, and proof-of-concept applications

Despite the limited number of studies on ICANS prediction, recent studies have supported the feasibility and growing potential of computational modeling.

#### 4.5.1 Multivariable risk score development

Several groups have proposed multivariable models that integrate baseline clinical factors, such as disease burden, performance status, and prior neurological conditions, with early post-infusion biomarkers, including peak ferritin, C-reactive protein (CRP), and interleukin-6 (IL-6). These models achieved encouraging discrimination during internal validation, with C-statistics ranging from 0.70 to 0.85. However, they are often limited by small sample sizes, lack of external validation, absence of temporal dynamics, and reliance on relatively simple statistical methods, such as logistic regression or Cox proportional hazards models.

#### 4.5.2 Mechanistic–machine learning hybrid models

Preliminary work combining mechanistic cytokine kinetic models with machine learning classifiers has shown improved predictive performance compared to clinical variables alone. These hybrid models provide greater biological interpretability by embedding mechanistic features within data-driven architectures and have the potential to simulate therapeutic interventions. However, clinical validation and large-scale applications are still lacking.

#### 4.5.3 Path toward clinical translation

Advancing from prototype development to clinical translation will require the coupling of modeling efforts with prospective biomarker cohort studies. Key requirements include standardized sampling protocols across institutions, consistent assay platforms, longitudinal sampling from pre-lymphodepletion to at least 30 days post-infusion, and detailed neurotoxicity grading using standardized criteria. Integrating multi-omics data, such as proteomic and transcriptomic profiles, could further refine the model accuracy. Open-access repositories containing harmonized biomarker datasets and computational models could accelerate field-wide progress, enable reproducibility, and facilitate benchmarking across independent research groups (162, 163).

### 4.6 Challenges, data gaps, and validation imperatives

Despite rapid methodological progress, several barriers limit the development and implementation of reliable prediction models for ICANS. These challenges reflect both the relative novelty of CAR T-cell therapy and the inherent complexity of capturing dynamic biological processes in critically ill patients. Systematic efforts to standardize data collection and establish shared repositories are essential to enable model development that achieves clinical utility and translational impact.

#### 4.6.1 Limited longitudinal biomarker datasets

The lack of comprehensive longitudinal biomarker datasets remains a major obstacle to robust modeling. Most studies capture only limited or single time-point data, which hinders the ability to define dynamic biological trajectories. Moreover, the specialized and resource-intensive nature of CAR T-cell therapy often limits the cohort size, compounding the challenges of developing and validating predictive models. To overcome this, future studies should emphasize dense temporal sampling during the early post-infusion period, extended follow-up for late events, and standardized collection protocols across different centers. Multi-institutional collaborations and registries can help pool data, while advanced statistical and data integration techniques can harmonize heterogeneous datasets.

#### 4.6.2 Heterogeneity in assays, protocols, and treatment regimens

Substantial variability exists in assay platforms, sampling timing, CAR T-cell constructs, lymphodepletion regimens, and supportive care practices. This heterogeneity complicates cross-study comparisons and limits generalizability.

Mitigation strategies include assay normalization, batch correction, and explicit modeling of treatment differences as covariates. Transfer learning approaches can adapt models trained on one CAR T product to others, while community-driven standards for biomarker measurement and toxicity grading would improve comparability and reproducibility.

#### 4.6.3 External validation

Most published models show strong performance in their development cohorts but demonstrate reduced generalizability and predictive accuracy when evaluated using independent external datasets. Robust external validation using independent datasets from different institutions, geographic regions, and CAR T-cell products is essential before clinical application. Prospective validation, in which predictions are made before outcomes occur, represents the benchmark for establishing model reliability and readiness for clinical use.

#### 4.6.4 Balancing complexity and interpretability

The central challenge in predictive modeling is balancing accuracy and interpretability. Complex machine learning models can achieve high predictive performance but often function as opaque “black boxes,” limiting clinician trust and regulatory approval. Clinically useful models should provide transparent reasoning that aligns with known pathophysiology and supports shared decision-making. Hybrid frameworks that integrate mechanistic knowledge with data-driven algorithms offer a balanced solution. Post-hoc interpretability tools such as SHAP values, feature importance rankings, or rule-based summaries can further enhance clinical transparency.

#### 4.6.5 Additional considerations

Clinical datasets often contain missing values and irregular sampling owing to real-world care variability. Approaches such as informed multiple imputations, probabilistic modeling, and time-series methods can mitigate these limitations.

Even well-validated models require practical integration into electronic health record systems and real-time clinical workflows. Automation of data ingestion, infrastructure for model execution, and clinician training in interpretation are essential for successful implementation. Models trained on demographically narrow populations may show uneven performance across patient subgroups. Systematic evaluation across age, race, ethnicity, and socioeconomic status should guide recalibration efforts to ensure equitable use and avoid bias in clinical decision-making (55, 135, 164–167).

#### 4.6.6 Clinical translation: pragmatic limitations and caveats

Despite rapid methodological progress, the path from a validated predictive model to a clinically actionable decision-support tool is fraught with pragmatic hurdles. First, there is a fundamental tension between model complexity and clinical utility. While hybrid mechanistic-machine learning models may achieve high accuracy, their “black-box” nature can erode clinician trust and complicate regulatory approval. For bedside use, models must provide interpretable, timely outputs that align with clinical intuition and workflow. Second, data latency presents a critical bottleneck. The predictive value of rapidly evolving cytokine or endothelial markers is nullified if assay turnaround times exceed the window for effective intervention. Real-time prediction requires the integration of rapid, point-of-care biomarker platforms, which are not yet standardized for ICANS management. Third, the generalizability of models trained on specific patient cohorts and CAR T-cell products remains questionable (1, 11, 13). A model derived from adult lymphoma patients receiving a CD19-directed 4-1BB CAR may not perform accurately in pediatric ALL patients or those receiving different constructs (e.g., CD28 co-stimulated CARs or those for solid tumors), due to differences in cytokine profiles, kinetics, and host biology (15). Finally, acting on probabilistic predictions introduces ethical and logistical challenges. Initiating prophylactic interventions like anakinra based on a model’s risk score, rather than overt symptoms, requires robust evidence of benefit and careful consideration of cost, added medication toxicity, and potential impact on CAR T-cell efficacy. Because of these issues, prospective studies that deliberately test biomarker-guided strategies will be essential. Only through these trials can we move from interesting predictions to safer, more effective care for patients.

## 5 Therapeutic and preventive strategies: mechanism-informed approaches

The transition from mechanistic understanding of ICANS to the development of targeted interventions illustrates the practical maturation of precision medicine (168). As targeted therapies are matched to oncogenic drivers, mechanistic understanding of ICANS enables preventive and therapeutic strategies to be mapped to distinct biological checkpoints during neurotoxicity development. This represents a shift from reactive management to a mechanism-informed prevention approach. Conventional management relied on overt symptoms such as confusion, aphasia and seizures, yet by the time these symptoms emerge, the pathological cascade has already progressed substantially (169). These mechanism-informed approaches are systematically categorized in Table 1, which maps each intervention to its targeted mechanistic step and summarizes the corresponding clinical evidence.

**Table 1:** Therapeutic & preventive strategies for ICANS: mechanism-informed approaches.

Strategy/intervention	Targeted mechanism	Clinical evidence & key findings
Established management		
Corticosteroids	Broad anti-inflammatory; reduces T-cell and myeloid cell activation and cytokine release.	Mainstay for severe ICANS/cerebral edema: Effective for symptom reversal, but concerns about attenuating CAR T-cell efficacy with early/high-dose use.
Tocilizumab (anti-IL-6R)	Blocks IL-6 signaling, a key driver of CRS.	Effective for CRS, limited for ICANS: Poor CNS penetration leads to a variable effect on neurotoxicity.
Anakinra (IL-1R antagonist)	Blocks IL-1 signaling, mitigating endothelial activation and neuroinflammation.	Gaining traction for treatment/prophylaxis: Early data shows reduced ICANS incidence/severity and preserved anti-tumor response.
IL-1 blockade (Anakinra)	Prophylactic blockade of IL-1 to prevent the cascade of endothelial activation and neuroinflammation.	Phase II/single-arm trials: Reduces severe ICANS rates while preserving CAR T-cell response rates. Under investigation in randomized trials.
GM-CSF inhibition (Lenzilumab)	Neutralizes GM-CSF to reduce activation of pro-inflammatory myeloid cells.	Preclinical & early clinical: Suggests reduction in CRS/ICANS without hindering CAR T function. Being tested in trials like ZUMA-19.
Endothelial stabilizers (Ang-Tie2 modulators)	Aims to strengthen vascular integrity by modulating the Ang-1 (protective)/Ang-2 (destabilizing) balance.	Conceptual/preclinical: Ang-2 is a promising biomarker and therapeutic target. Translational work is needed.
Blood-CNS barrier protectants (MMP inhibitors, S1P modulators, etc.)	Protects blood–brain barrier integrity and provides neuroprotection against excitotoxicity/oxidative stress.	Plausible but preclinical: Requires validation in relevant ICANS models.
Safety switches (iCasp9, switchable CARs)	Provides a “kill switch” or a way to modulate CAR T-cell activity to blunt excessive activation.	Engineering approach: Adds an upstream safety layer. Effectiveness depends on integration with biomarker monitoring for timely activation.
Monitoring & dosing systems		
Closed-loop/adaptive dosing	Integrates real-time biomarker data (cytokines, endothelial markers, EEG) to automatically guide interventions.	Pilot feasibility stage: A conceptual framework that requires studies to test safety, logistics, and effectiveness.

### 5.1 Established management: corticosteroids, tocilizumab, anakinra

Corticosteroids remain the primary therapeutic option for severe ICANS, particularly when cerebral edema is present (170). High-dose dexamethasone at 10 mg intravenously every 6 h produces rapid neurologic improvement within hours in the majority of patients, with response rates exceeding 80% documented across multiple CAR T-cell products (171). This consistent efficacy has established corticosteroids as standard therapy for grade 3 or higher neurotoxicity. Concerns persist regarding potential suppression of CAR T-cell expansion and antitumor activity, though accumulating clinical evidence suggests that brief corticosteroid courses of 3 to 7 days with rapid taper have minimal impact on tumor response rates (172). Several centers have begun implementing earlier corticosteroid intervention at the onset of grade 1 or 2 ICANS to prevent progression, and preliminary observations suggest this approach may reduce severe neurotoxicity without compromising therapeutic efficacy (173).

Tocilizumab, the anti-IL-6 receptor monoclonal antibody that transformed CRS management, demonstrates limited efficacy in ICANS and may be associated with increased neurotoxicity severity in some patients (8, 83, 174, 175).

### 5.2 Mechanistically targeted prophylaxis

Beyond established agents, several mechanism-based interventions aim to intercept the neurotoxic cascade at earlier checkpoints, with specific targets and levels of clinical validation, as detailed in Table 1. Prophylactic anakinra in single-arm and early phase trials reduced severe ICANS rates with preserved response rates and is under investigation in randomized and multicenter studies (176–178). GM-CSF has emerged as another attractive prophylactic target. Preclinical and early clinical studies suggest that GM-CSF neutralization reduces myeloid activation, CRS and neurotoxicity without hindering CAR function, and early trials such as ZUMA-19 have incorporated lenzilumab prophylaxis into their design (179–183). Endothelial stabilization represents another conceptually appealing approach. Therapies aimed at strengthening endothelial junctions or modulating the angiopoietin-1 to angiopoietin-2 balance could theoretically prevent the vascular leak that permits cytokine entry into the central nervous system. However, these approaches remain largely preclinical, and angiopoietin-2, as both a biomarker and therapeutic target, merits a translational focus (83, 184–187). Similarly, direct blood-CNS barrier protection through matrix metalloproteinase inhibitors or sphingosine-1-phosphate receptor modulators, along with neuroprotective agents like antioxidants and anti-excitotoxic compounds, hold mechanistic logic but require preclinical validation in appropriate ICANS models (4, 188).

### 5.3 Engineering “safety switches” in CAR designs

An alternative strategy addresses toxicity at its source by directly building control mechanisms into the CAR construct. Inducible suicide switches, such as iCasp9, allow for rapid elimination of CAR T-cells when toxicity becomes unmanageable. CARs that respond to small molecule triggers or incorporate graded activation can modulate activity more precisely, blunting excessive cytokine release while preserving some therapeutic function (4, 189). Such designs allow for direct modulation of CAR activity when toxic signals emerge. Combined with biomarker surveillance, this could enable earlier intervention based on individual inflammatory patterns.

### 5.4 Closed loop/adaptive dosing and monitoring

**Figure 3:**
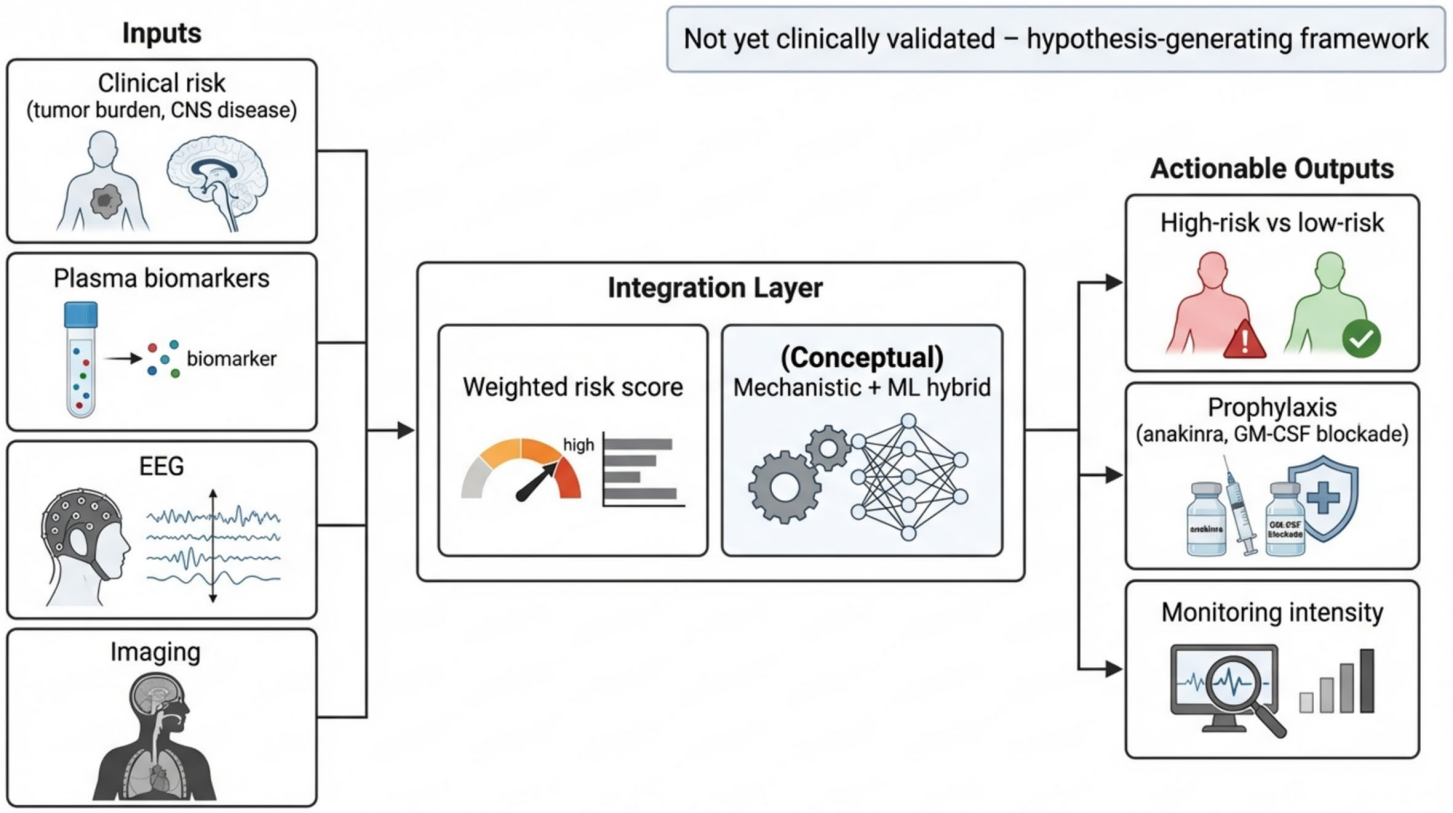
Conceptual multimodal framework for ICANS risk integration. This schematic illustrates a integrates clinical risk factors, plasma biomarkers, EEG findings, and imaging into a weighted risk score using a hybrid mechanistic and machine-learning approach. The intent is to support potential clinical decisions such as identifying high- versus low-risk patients, adjusting monitoring intensity, and exploring prophylactic strategies in future studies. It is presented to highlight how multimodal biomarkers could eventually inform risk-guided ICANS management.

Engineered safety switches and emerging biomarkers could potentially be integrated into closed-loop systems that connect real-time monitoring with therapeutic intervention (24). Such systems would integrate real-time data from cytokine sensors, point-of-care endothelial markers, and electroencephalography with predefined algorithms to guide intervention. Based on evolving inflammatory or neurologic parameters, these algorithms can modulate infusion kinetics, initiate prophylactic therapy, or activate engineered safety switches before toxicity progresses. This approach would shift management from reactive treatment to preemptive control, although practical implementation faces substantial challenges. Pilot feasibility studies are needed to establish safety, assess logistical requirements, and determine whether adaptive interventions improve clinical outcomes compared to standard protocols (83, 190, 191). This integrative monitoring and dosing concept is summarized in the final section of Table 1. Collectively, these modeling approaches highlight the need for a continuous feedback system that bridges biomarker discovery, predictive computation, and clinical applications. To extend these mechanistic and biomarker insights toward potential clinical application, we present a conceptual framework for multimodal ICANS risk integration (Figure 3). In this framework, clinical risk factors, plasma biomarkers, EEG findings, and neuroimaging results are combined within a weighted integration layer to generate a provisional risk estimate that could inform monitoring intensity and consideration of prophylactic strategies. This model is hypothesis-generating and is not yet clinically validated, but it illustrates how convergent data streams may ultimately support safer implementation of CAR T-cell therapy.

## 6 Knowledge gaps, challenges and future directions

Despite substantial progress in understanding the mechanisms of ICANS, the clinical application of these insights remains incomplete. Biomarkers with sufficient predictive accuracy and temporal resolution to guide intervention are still needed, and whether prophylactic strategies can preserve antitumor efficacy while reducing toxicity has not been conclusively demonstrated. Prospective trials are essential to establish whether mechanistic knowledge translates to improved therapeutic outcomes. The temporal hierarchy of cytokine, endothelial, cerebrospinal, and neurophysiological biomarkers, summarized in Figure 2, provides a framework for prioritizing future multimodal studies. By delineating predictive, diagnostic, and recovery windows, this model emphasizes when biomarker sampling and intervention are most biologically informative.

### 6.1 Gaps in human data

Large prospective studies that combine serial measurements across plasma, cerebrospinal fluid, electroencephalography and neuroimaging are limited, hampering efforts to validate multimodal biomarker profiles with clinically useful predictive power. Neuropathological data remain scarce, restricting our ability to link proposed mechanisms to actual tissue pathology in affected brain regions. These deficits hinder the development of robust risk stratification approaches and the identification of the mechanistic steps that are the most viable targets for intervention (55, 192). These challenges, along with the subsequent issues and recommended solutions, are systematically summarized in Table 2.

**Table 2:** Knowledge gaps, challenges, and future directions in ICANS research.

Category	Key issues & challenges	Proposed solutions & future directions
Gaps in human data	Limited large, prospective multimodal biomarker cohorts (plasma, CSF, EEG, MRI). Scarce neuropathology data to validate mechanistic hypotheses.	Establish large-scale studies that systematically collect serial bio-samples and neurodiagnostic data. Prioritize neuropathological studies from consented patients.
Standardization issues	Assay variability, heterogeneous sampling schedules, and different ICANS grading schemas prevent data pooling and robust modeling.	Develop consensus recommendations for standardized sampling timepoints, assay methods, and data units. Promote use of centralized labs.
Cross-trial heterogeneity	Different CAR constructs, disease contexts, and clinical practices create variable toxicity profiles, hindering comparison and generalizability.	Stratify data analysis by CAR construct (e.g., CD28 vs. 4-1BB) and meticulously record clinical metadata in trials and studies.
Differential diagnosis of neurotoxicity	The pathophysiological and clinical distinction between ICANS (diffuse BBB dysfunction) and other syndromes like Tumor Inflammation-Associated Neurotoxicity (TIAN; focal, tumor-centric inflammation) is not always clear but is critical for management. Biomarkers and models for ICANS may not apply to TIAN.	1. Develop and validate syndrome-specific diagnostic criteria and biomarker panels. 2. In trials involving CNS-directed therapies, design separate monitoring and intervention protocols for ICANS vs. TIAN. 3. Encourage systematic reporting of neurotoxicity subtype in clinical studies to build distinct knowledge bases.
Ethical & logistical challenges	Burden on patients from frequent CSF sampling and repeated neurodiagnostic tests (MRI/EEG), alongside high costs.	Develop and validate non-invasive surrogates (e.g., plasma proteomics, remote EEG). Balance scientific rigor with patient welfare in trial design.
Integration with other toxicities	ICANS shares inflammatory pathways with CRS and other organ toxicities (e.g., cardiotoxicity), creating competing risks and covarying endpoints.	Develop integrated models that account for multiple toxicities simultaneously. Design trials with co-primary or composite endpoints.
Key recommendations	A need for coordinated, transparent, and biomarker-driven research to accelerate progress.	1. Standardize: Adopt harmonized biomarker panels and fixed sampling timepoints. 2. Collaborate: Create multi-site consortia for powerful multimodal studies. 3. Validate: Pre-register analysis plans and share data for reproducibility. 4. Translate: Conduct randomized, biomarker-guided trials of prop

### 6.2 Standardization issues

Different assay platforms for cytokines and endothelial markers produce inconsistent results with ELISA, multiplex arrays, and electrochemiluminescence methods yielding values that cannot be directly compared (193). Sample handling varies considerably between centers in terms of processing time, storage conditions, and freeze–thaw cycles, all of which affect the stability of analytes. ICANS grading has also lacked uniformity, with studies using CTCAE, ASTCT, or CARTOX criteria that define severity thresholds differently (194). This variability complicates efforts to validate findings across cohorts and develop predictive models with external applicability. Standardized protocols for sample collection, processing, and measurement would facilitate cross-institutional comparisons and accelerate identification of reliable biomarker signatures.

### 6.3 Cross-trial heterogeneity and comparability

Toxicity patterns vary markedly across CAR T-cell studies because of differences in product design and clinical context. Constructs incorporating CD28 co-stimulation typically produce earlier and more intense CRS and ICANS than those using 4-1BB domains, reflecting distinct activation kinetics and cytokine profiles (195). Patient populations also differ in ways that influence the risk of toxicity. High tumor burden, prior lines of therapy, baseline organ dysfunction and disease histology modify inflammatory responses to CAR T-cell expansion (196). Conditioning chemotherapy regimens range from aggressive lymphodepletion to reduced-intensity preparative regimens, each creating different cytokine environments before infusion (197). Institutional practices for toxicity management have shifted over time, with thresholds for corticosteroid administration, intensive care transfer and varying prophylactic interventions (162). Biomarker studies conducted in a single trial setting may not be generalizable to products with different biological properties or patient populations with distinct baseline characteristics. Developing broadly applicable risk models requires explicit stratification by CAR construct and comprehensive documentation of clinical variables that modify toxicity susceptibility (55, 198–201).

### 6.4 Ethical and logistical challenges

Comprehensive biomarker collection faces significant practical and ethical challenges. Patients with evolving neurotoxicity often cannot provide informed consent for serial invasive procedures, and families may reasonably question the efficacy of lumbar punctures that offer no direct therapeutic benefit (3). Moving critically ill patients to imaging suites introduces risk, and completing MRI protocols becomes difficult when patients are confused or agitated. Financial realities compound these challenges. Centers performing fewer than 20 CAR infusions per year typically lack infrastructure for specimen processing, dedicated research coordination or continuous neurophysiology monitoring (202). As a result, noninvasive alternatives such as plasma proteomics and portable EEG systems could expand participation beyond specialized centers, though their clinical validity is relative to cerebrospinal fluid analysis and formal neurophysiology assessment requires demonstration (203, 204).

### 6.5 Integration with other toxicity modalities

ICANS cannot be fully understood in isolation from the broader toxicity spectrum of CAR T-cell therapy. CRS, cardiac dysfunction, hepatic injury, and coagulopathy emerge from overlapping inflammatory pathways characterized by cytokine dysregulation, myeloid cell activation and endothelial disruption (205, 206). The neuro-cardiac axis deserves particular attention, as both organ systems are vulnerable to the same inflammatory mediators through parallel mechanisms of immune-mediated injury (205). Clinically, these toxicities are interconnected rather than independent. Severe CRS commonly precedes ICANS, suggesting a temporal relationship where systemic inflammation primes the central nervous system for subsequent injury (4, 207).

### 6.6 Recommendations for future studies

Addressing these challenges requires coordinated action across multiple fronts. Coordinating measurement approaches across institutions is a practical first step. Consensus time points at baseline, immediately after infusion, 24 h, 72 h, day 7, and day 14 would create comparable longitudinal profiles. Standardizing assays through shared platforms addresses the technical inconsistencies that complicate current comparisons. Most centers lack sufficient patient volume to generate the sample sizes required for multimodal model development. Consortia that combine imaging, electroencephalography, cerebrospinal fluid, and plasma data across multiple sites would provide the statistical power needed to train and validate predictive algorithms. Independent validation in separate cohorts distinguishes reproducible biomarker signatures from misleading ones. Finally, mechanistic insights must be translated into interventional trials. Biomarker-guided studies testing prophylactic strategies such as anakinra or GM-CSF blockade should incorporate predefined decision rules for dose modification or treatment initiation, with neurocognitive outcomes measured systematically rather than relying solely on clinically graded toxicity scales (208). The accompanying Table 2 provides a consolidated overview of these knowledge gaps, challenges, and the proposed strategic recommendations for the field.

## 7 Conclusions and future perspectives

ICANS pathogenesis follows a predictable sequence from systemic immune activation through endothelial injury and blood–brain barrier disruption to CNS inflammation and neuronal dysfunction. Early plasma markers of cytokine surge and endothelial activation offer a window for preemptive intervention before clinical neurotoxicity manifests. Emerging prophylactic strategies, particularly IL-1 blockade with anakinra and GM-CSF neutralization, demonstrate feasibility of toxicity mitigation without compromising antitumor efficacy. Translating this mechanistic knowledge into clinical decision support requires hybrid computational frameworks that combine mechanistic ODE systems with machine learning algorithms. These predictive models can integrate temporal cytokine profiles, endothelial markers, imaging abnormalities, and EEG signatures to enable real-time risk stratification. Model development and validation require longitudinal multimodal datasets, which remain scarce. Multi-institutional consortia with standardized sampling protocols and shared data repositories are essential to address this gap and enable external validation across independent cohorts and CAR T-cell products in the future. Future studies must also differentiate the mechanistic and predictive frameworks for ICANS from other neuroinflammatory sequelae of immunotherapy, such as Tumor Inflammation-Associated Neurotoxicity (TIAN), to ensure precise and effective management across the expanding spectrum of cellular therapies. Moreover, future studies should focus on biomarker-guided clinical trials. Randomized studies testing prophylactic interventions such as IL-1 blockade, GM-CSF neutralization, or endothelial stabilizers in high-risk patients identified through predictive algorithms will determine whether computational models translate into improved outcomes. Pre-specified modeling challenges with benchmark datasets would accelerate algorithm refinement and establish reproducibility across platforms.

Ultimately, the path to safer CAR T-cell therapy depends on identifying patients at risk before the onset of symptoms and intervening with precision. The mechanistic and predictive frameworks provide the foundation for this approach. Preventing ICANS without compromising efficacy will extend the transformative reach of CAR T-cell therapy while reducing the burden of severe neurotoxicity that limits its broader application.
